# Preoperative diagnostic criteria for scleroatrophic gallbladder: A systematic review protocol

**DOI:** 10.1371/journal.pone.0300336

**Published:** 2024-03-13

**Authors:** Renato Bessa-Melo, Cristina Fernandes, Ana Aguiar, Santiago Lopez-Ben, Luís Guimarães, Pedro Serralheiro

**Affiliations:** 1 Faculty of Health Sciences, University of Beira Interior, Covilhã, Portugal; 2 General Surgery, ULS Cova da Beira, Covilhã, Portugal; 3 General Surgery, Centro Hospitalar Universitário São João, Porto, Portugal; 4 Faculty of Medicine, University of Porto, Porto, Portugal; 5 EPIUnit—Institute of Public Health, University of Porto, Porto, Portugal; 6 Laboratory for Integrative and Translational Research in Population Health (ITR), University of Porto, Porto, Portugal; 7 ICBAS—Instituto de Ciências Biomédicas Abel Salazar, University of Porto, Porto, Portugal; 8 Hepatobiliary and Pancreatic Surgery Unit, Department of Surgery, Dr. Josep Trueta Hospital, Girona, Spain; 9 Imaging Department Centro Hospitalar Universitário São João, Porto, Portugal; 10 General Surgery, Centro Hospitalar Universitário de Coimbra, Coimbra, Portugal; 11 Faculty of Medicine, University of Coimbra, Coimbra, Portugal; Hacettepe University: Hacettepe Universitesi, TURKEY

## Abstract

**Background:**

Although scleroatrophic gallbladder is a rare condition, it presents significant clinical challenges in diagnosis and management. More agreement is needed on this disorder’s diagnostic criteria and optimal management approach. We will conduct a systematic review to summarise the scleroatrophic gallbladder’s preoperative diagnostic criteria, including imaging modalities.

**Methods:**

A systematic review will be undertaken using the PRISMA guidelines. The protocol has been registered in PROSPERO (CRD42024503701). We will search in Medline (via PubMed), Embase, SCOPUS, the Cochrane Library, and Web of Science to find original studies reporting about scleroatrophic gallbladder or synonymous. Two reviewers will independently screen the titles and abstracts following the eligibility criteria. We will include all types of studies that describe any diagnostic criteria or tools. After retrieving the full text of the selected studies, we will conduct a standardised data extraction. Finally, a narrative synthesis will be performed. The quality of the identified studies will be assessed using the Quality Assessment of Diagnostic Accuracy Studies- 2 tool.

**Discussion:**

This systematic review will provide information on the preoperative diagnostic criteria of the scleroatrophic gallbladder and the value of imaging studies in its diagnosis. In addition, this work will aid doctors in the decision-making process for diagnosing scleroatrophic gallbladder and propose treatment approaches to this condition.

**Systematic review registration:**

The protocol has been registered in PROSPERO (CRD42024503701).

## Introduction

The scleroatrophic gallbladder is referred to as a condition when the surgeon encounters a shrunken gallbladder with deformation of the hepatic porta, typically resulting from chronic inflammation and scarring. There are no known preoperative gold standard tools in the diagnosis of this condition. Histology reports of surgical specimens from cholelithiasis cholecystectomies show that scleroatrophic alterations can occur in about 1.2% of the patients [1,2]. Despite its rarity, scleroatrophic gallbladder poses clinical challenges in diagnosis and management. Additional consensus regarding this condition’s preoperative diagnostic criteria, including its imaging modalities, is required. There are literature references to other conditions, such as contracted gallbladder, shrunken gallbladder, and fibrous gallbladder, with ambiguous definitions [3–6] that may be assumed as synonymous with scleroatrophic gallbladder. In addition, it is associated with a higher risk of bile duct injury and conversion to open surgery [7].

Scleroatrophic gallbladder is often asymptomatic and can present diagnostic challenges due to its nonspecific clinical manifestations. It is associated with chronic cholecystitis, and its exact aetiology is poorly understood [7]. Its management remains controversial, with varying recommendations between surgical intervention and more conservative management.

Cholecystectomy is the optimal management of cholelithiasis-related pathology as it removes both the gallstones and the gallbladder, preventing recurrent disease. The only common side effect following gallbladder removal is an increased stool frequency, which is clinically significant in less than 5% of patients [8]. Since its introduction in the late’80s, laparoscopic cholecystectomy has been adopted rapidly. It is one of the most common surgical procedures performed worldwide, with a low conversion rate to open cholecystectomy (<0.5%) in specialised centres [9]. The main drawback of the laparoscopic technique has been a slightly higher incidence of common hepatic or bile duct injury (0.2–0.4% vs 0.1% for open cholecystectomy). In a “difficult gallbladder”, these numbers can change, and the conversion rate could be as high as 30%, whereas the iatrogenic injuries could be as high as 0.6% [10]. The scleroatrophic gallbladder is one of the conditions that may be associated with a “difficult gallbladder” and with an increased risk of bile duct injury and conversion to open surgery [11]. It is then necessary to clarify the best preoperative diagnostic criteria to adopt appropriate therapeutic strategies and assist clinicians to understand better this condition’s clinical features, diagnostic challenges, and management options and guide future research and clinical practice.

This systematic review aims to assess the diagnostic criteria and accuracy of preoperative diagnostic criteria and tools developed to identify scleroatrophic gallbladder. It will also seek to identify which diagnostic methods are currently used and the quality and heterogeneity of the studies where these methods were described.

A preliminary search found no previous systematic reviews investigating the development or validation of preoperative diagnostic criteria for scleroatrophic gallbladder.

### Objectives

#### Primary objective

To identify and describe preoperative diagnostic criteria and tools developed for scleroatrophic gallbladder diagnosis.

#### Secondary objectives

- To determine what methods of preoperative diagnosis have been used.
- To assess the quality and source of heterogeneity of the studies where the diagnostic criteria or tools were described.

## Materials and methods

### Review question

What are the preoperative diagnostic criteria for scleroatrophic gallbladder in patients submitted to cholecystectomy?

We used the acronym PIT instead of PICO to develop the review question, as suggested in the Cochrane Handbook for Systematic Reviews of Diagnostic Test Accuracy [12]. The three letters refer to Population, Index test(s), and Target condition.

*Population*–The systematic review will include studies of adults (age 18 or more) submitted to cholecystectomy with a scleroatrophic gallbladder.

*Index test*–The index test is any attempt to preoperatively diagnose a scleroatrophic gallbladder using either a scoring system or diagnostic criteria, mainly based on imaging studies. Any attempt to confirm the preoperative diagnosis of a scleroatrophic gallbladder will be used as a reference standard.

*Target condition*–Scleroatrophic gallbladder (or synonyms identified by the team) is the target condition for this review.

### Design

We will systematically review studies reporting preoperative diagnostic criteria for scleroatrophic gallbladder. This protocol has been written in line with the Preferred Reporting Items for Systematic Review and Meta-Analysis Protocols (PRISMA-P) checklist [13,14]. In addition, it has been registered with PROSPERO (CRD42024503701). The review will be conducted and reported following the PRISMA guidelines [15]. Any protocol amendment will be noted in the systematic review publication.

### Eligibility criteria

Prospective and retrospective studies, trials, and clinical cases, including patients with scleroatrophic gallbladder or synonymous, which present preoperative diagnostic criteria or tools for diagnosis, will be eligible. There will be no restrictions concerning the year of publication, geographical setting or language.

### Information sources

We will search in Medline (via PubMed), Embase, the Cochrane Library, SCOPUS, Web of Science, and Google Scholar to identify all eligible studies. The references of the selected studies and conference abstracts will be checked to find different eligible studies. Any additional potentially eligible studies identified by Hepato-Pancreatico-Biliary (HPB) experts in our team will be screened. References lists in included studies and any relevant systematic review identified will be checked to find other studies that might be eligible for the review. References will be exported into the ZOTERO software, and duplicates will be removed.

### Search strategy

The search will include combinations of keywords and Medical Subjects Headings (MeSH) terms for ‘gallbladder’, ‘scleroatrophic’, ‘difficult’, ‘contracted’, ‘atrophic’ and ‘fibrosis’. S1 Appendix provides the search strategy developed for MEDLINE. An information specialist and an HPB expert in our team were consulted to ensure the completeness of our search strategy.

### Study selection

Two authors will screen the references, firstly based on the titles and abstracts. Then, the full texts of references deemed potentially eligible will be assessed independently for inclusion by two reviewers. If there is any disagreement, a third reviewer will be involved. All potentially eligible articles in languages other than English, French, Spanish and Portuguese will be translated into English for eligibility assessment. A PRISMA flow diagram will document the excluded references and reasons for full-text exclusions. To perform all phases of the study selection, we will use the Rayyan® application for systematic reviews [16].

#### Data extraction and synthesis

Two independent reviewers will conduct the data extraction. A standardised extraction form (S2 Appendix) will be piloted in five studies, after which adjustments will be made as needed. A third reviewer will be consulted in case any disagreements would arise. In addition, the authors of the original studies will be contacted in case of ambiguity in their reported data. We will extract the characteristics of the study (year, location, design, sample size), the features of the participants, the definition and diagnostic criteria of the scleroatrophic gallbladder and the diagnostic methods used. Final data will be extracted from the included studies into an Excel spreadsheet.

As we anticipate substantial heterogeneity across the included studies, we will not conduct a meta-analysis or a meta-synthesis. Instead, a narrative synthesis of the extracted data will be performed according to the methods described by Popay et al. [17]. This method is described by some authors as a textual narrative synthesis and is characterised by having a standard data extraction in contrast with a simple narrative synthesis [18]. In addition, this method is used in systematic reviews by several authors [19,20].

It includes several stages:

1. A preliminary synthesis, which involves a descriptive summary of the extracted information on study characteristics, findings, and critical appraisal.
2. The relationships and associations between study characteristics and reported findings within individual and across studies will be explored. In addition, the nature of heterogeneity in the investigations regarding variability in study populations, study designs and settings and their influence will be investigated during this stage.
3. Discussion of the findings, their implications and the provision of recommendations for future research and clinical practice.

### Risk of bias and quality assessment

To evaluate the methodological quality, two independent reviewers will assess all studies using signalling questions in the Quality Assessment of Diagnostic Accuracy Studies-2 tool (QUADAS-2) [21], and a third reviewer will resolve eventual disagreements. The reviewers will assess the signalling question as ‘unclear’ if the study did not provide the information. For each domain, studies will be judged as ‘low risk’ if all signalling questions were answered ‘yes’, ‘high risk’ if the answer to at least one signalling question was ‘no’, or ‘unclear’ in all other cases. Randomised controlled trials are initially considered high quality, while observational data starts at a low-quality level due to potential residual confounding [21].

### Ethics

This protocol will not evaluate individual patient information or affect patient rights and, therefore, does not require ethical approval.

## Discussion

This systematic review investigates the preoperative diagnostic criteria of the scleroatrophic gallbladder. To our knowledge, this systematic review is the first that will present the synthesis of evidence on preoperative diagnostic criteria for scleroatrophic gallbladder.

### Expected benefits

This review will assist doctors in the process of diagnosing a scleroatrophic gallbladder, therapeutic decision-making, properly informing patients, and promoting a shared decision that is a fundamental part of patient-centred care.

Our review will be based on an exhaustive search strategy through various reference databases, which will therefore provide an evidence synthesis on diagnostic criteria for scleroatrophic gallbladder and the tools used in its diagnosis. Thus, it will enable us to identify gaps and avenues for future research and improve practices.

### Limitations

We do expect a high heterogeneity between the different studies. There may be a limited number of studies available in the literature. The lack of research on this topic could affect the depth and breadth of the evidence base for the systematic review.

### Dissemination

Results from this review will be disseminated through peer-reviewed journals and conference reports.

## Supporting information

S1 Checklist PRISMA-P (Preferred Reporting Items for Systematic Review and Meta-Analysis Protocols) 2015 checklist: Recommended items to address in a systematic review protocol. The PRISMA-P 2015 checklist for items to address in a systematic review protocol.
(DOCX)

S1 Appendix MEDLINE search strategy. (DOCX)

S2 Appendix Data extraction form. (DOCX)

## References

[1] Meirelles-Costa ALA, Bresciani CJC, Perez RO, Bresciani BH, Siqueira SAC, Cecconello I. Are histological alterations observed in the gallbladder precancerous lesions? Clinics. 2010 Feb;65(2):143–50. doi: 10.1590/S1807-59322010000200005 20186297 PMC2827700

[2] Holanda AKG, Lima Júnior ZB. Gallbladder histological alterations in patients undergoing cholecystectomy for cholelithiasis. Rev Colégio Bras Cir. 2019;46(6):e20192279. 10.1590/0100-6991e-20192279 31967243

[3] Tongyoo A, Chotiyasilp P, Sriussadaporn E, Limpavitayaporn P, Mingmalairak C. The pre-operative predictive model for difficult elective laparoscopic cholecystectomy: A modification. Asian J Surg. 2021 Apr;44(4):656–61. doi: 10.1016/j.asjsur.2020.11.018 33349555

[4] Hanson-Viana E, Ayala-Moreno EA, Ortega-Leon LH, Montalvo-Javé EE. The Association of Preoperative Risk Factors for Laparoscopic Conversion to Open Surgery in Elective Cholecystectomy. Euroasian J Hepato-Gastroenterol. 2022 Jul 13;12(1):6–9. doi: 10.5005/jp-journals-10018-1366 35990867 PMC9357520

[5] Anderson K, Roland AL, Miller MP, Foretia DA. Beware of the shrunken gallbladder–Case report of intraoperatively diagnosed gallbladder agenesis. Int J Surg Case Rep. 2022 Sep;98:107588. doi: 10.1016/j.ijscr.2022.107588 36058154 PMC9482971

[6] Iwashita Y, Hibi T, Ohyama T, Umezawa A, Takada T, Strasberg SM, et al. Delphi consensus on bile duct injuries during laparoscopic cholecystectomy: an evolutionary cul-de-sac or the birth pangs of a new technical framework? J Hepato-Biliary-Pancreat Sci. 2017 Nov;24(11):591–602. doi: 10.1002/jhbp.503 28884962

[7] Akoglu M, Ercan M, Bostanci EB, Teke Z, Parlak E. Surgical outcomes of laparoscopic cholecystectomy in scleroatrophic gallbladders. Turk J Gastroenterol. 2010 Jun 1;21(2):156–62. doi: 10.4318/tjg.2010.0075 20872330

[8] Beckingham IJ. ABC of diseases of liver, pancreas, and biliary system. Gallstone disease. BMJ. 2001 Jan 13;322(7278):91–4. doi: 10.1136/bmj.322.7278.91 11154626 PMC1119388

[9] Nassar AHM, Zanati HE, Ng HJ, Khan KS, Wood C. Open conversion in laparoscopic cholecystectomy and bile duct exploration: subspecialisation safely reduces the conversion rates. Surg Endosc. 2022 Jan;36(1):550–8. doi: 10.1007/s00464-021-08316-1 33528666 PMC8741693

[10] Hussain A. Difficult Laparoscopic Cholecystectomy: Current Evidence and Strategies of Management. Surg Laparosc Endosc Percutan Tech. 2011;21(4). doi: 10.1097/SLE.0b013e318220f1b1 21857467

[11] Gigot JF, Etienne J, Aerts R, Wibin E, Dallemagne B, Deweer F, et al. The dramatic reality of biliary tract injury during laparoscopic cholecystectomy: An anonymous multicenter Belgian survey of 65 patients. Surg Endosc. 1997 Dec;11(12):1171–8. 9373288 10.1007/s004649900563

[12] Leeflang MM, Davenport C, Bossuyt PM. Chapter 5: Defining the review question. Draft version (4 October 2022). In: Deeks J, Bossuyt P, Leeflang M, Takwoingi Y, editors. Cochrane Handbook for Systematic Reviews of Diagnostic Test Accuracy Version 2 [Internet]. London: Cochrane; Available from: https://training.cochrane.org/handbook-diagnostic-test-accuracy

[13] PRISMA-P Group, Moher D, Shamseer L, Clarke M, Ghersi D, Liberati A, et al. Preferred reporting items for systematic review and meta-analysis protocols (PRISMA-P) 2015 statement. Syst Rev. 2015 Dec;4(1):1. doi: 10.1186/2046-4053-4-1 25554246 PMC4320440

[14] Page MJ, Moher D, Bossuyt PM, Boutron I, Hoffmann TC, Mulrow CD, et al. PRISMA 2020 explanation and elaboration: updated guidance and exemplars for reporting systematic reviews. BMJ. 2021 Mar 29;n160. doi: 10.1136/bmj.n160 33781993 PMC8005925

[15] Moher D, Liberati A, Tetzlaff J, Altman DG. Preferred Reporting Items for Systematic Reviews and Meta-Analyses: The PRISMA Statement. 7. PMC3090117 21603045

[16] Ouzzani M, Hammady H, Fedorowicz Z, Elmagarmid A. Rayyan—a web and mobile app for systematic reviews. Syst Rev. 2016 Dec 5;5(1):210. doi: 10.1186/s13643-016-0384-4 27919275 PMC5139140

[17] Popay J, Roberts H, Sowden A, Petticrew M, Arai L, Rodgers M, et al. Guidance on the conduct of narrative synthesis in systematic reviews. Prod ESRC Methods Programme Version. 2006;1(1):b92.

[18] Xiao Y, Watson M. Guidance on Conducting a Systematic Literature Review. J Plan Educ Res. 2019 Mar;39(1):93–112.

[19] Aiyegbusi OL, Macpherson K, Elston L, Myles S, Washington J, Sungum N, et al. Patient and public perspectives on cell and gene therapies: a systematic review. Nat Commun. 2020 Dec 8;11(1):6265. doi: 10.1038/s41467-020-20096-1 33293538 PMC7722871

[20] Jessop ZM, Dobbs TD, Ali SR, Combellack E, Clancy R, Ibrahim N, et al. Personal protective equipment for surgeons during COVID-19 pandemic: systematic review of availability, usage and rationing. Br J Surg. 2020 Aug 24;107(10):1262–80. doi: 10.1002/bjs.11750 32395837 PMC7273092

[21] Whiting PF. QUADAS-2: A Revised Tool for the Quality Assessment of Diagnostic Accuracy Studies. Ann Intern Med. 2011 Oct 18;155(8):529. doi: 10.7326/0003-4819-155-8-201110180-00009 22007046

