# Supplementary material for: Preoperative diagnostic criteria for scleroatrophic gallbladder: A systematic review protocol
Source: PLoS One. 2024 Mar 13;19(3):e0300336. doi: 10.1371/journal.pone.0300336 (PMC10936762; doi:10.1371/journal.pone.0300336)
Supplement: S1 Appendix — (DOCX) [file pone.0300336.s002.docx]

**S2 Appendix**

**Medeline search strategy**

[Gallbladder[MeSH Major Topic] AND (scleroatrophic OR contracted OR shrunken OR fibrous OR difficult OR atrophic OR fibrosis) AND (disease* OR pathology OR physiopathology OR complication* OR classification OR diagnosis OR imaging OR surgery)]
