# Supplementary material for: Preoperative diagnostic criteria for scleroatrophic gallbladder: A systematic review protocol
Source: PLoS One. 2024 Mar 13;19(3):e0300336. doi: 10.1371/journal.pone.0300336 (PMC10936762; doi:10.1371/journal.pone.0300336)
Supplement: S2 Appendix — (DOCX) [file pone.0300336.s003.docx]

**S3 Appendix**

**Data extraction form**

**Study Identification**

Last Name of First Author

Year

PMID

Other ID

Publication Type

Full report

Abstract

Letter

Other (specify)

**Eligibility**

Review Inclusion Criteria

Yes

No

Unclear

Type of Study

Guideline

Review

Randomised trial

Non-randomised trial

Cohort

Case report

Other (specify)

Participants

Age > 18

Yes

No

Not specified

Gender

Both

Male

Female

Not specified

Number patients included

Type of Intervention

Surgery

Open

Laparoscopic

Both

Non-operative management

Types of outcome measures

Complications

Biliary lesions

Conversion to open

Other (specify)

Reason for Exclusion

**Data**

Scleroathrofic gallbladder

Yes

No

Other

Specify

Definition (open text)

Index teste

Preoperative

Yes

No

Image technique

Yes

No

Single

Multiple

Which?

Ultrasonography

CT scan

MRI

Other (specify)

Criteria (open text)

Other

Specify

Criteria

Intraoperative

Yes

No

Criteria (open text)

Postoperative

Yes

No

Histopathology

Yes

No

Other (specify)

Criteria (open text)

Comparator test

Specify

Criteria

**Risk of bias assessment (QUADAS-2)**

**DOMAIN 1: PATIENT SELECTION**

1. Risk of Bias

Describe methods of patient selection:

Was a consecutive or random sample of patients enrolled?

Yes/No/Unclear

Was a case-control design avoided?

Yes/No/Unclear

Did the study avoid inappropriate exclusions?

Yes/No/Unclear

Could the selection of patients have introduced bias?

RISK: LOW/HIGH/UNCLEAR

1. Concerns regarding applicability

Describe included patients (prior testing, presentation, intended use of index test and setting):

Is there concern that the included patients do not match the review question?

CONCERN: LOW/HIGH/UNCLEAR

**DOMAIN 2: INDEX TEST(S)**

If more than one index test was used, please complete for each test.

1. Risk of Bias

Describe the index test and how it was conducted and interpreted:

Were the index test results interpreted without knowledge of the results of the reference standard?

Yes/No/Unclear

If a threshold was used, was it pre-specified?

Yes/No/Unclear

Could the conduct or interpretation of the index test have introduced bias?

RISK: LOW /HIGH/UNCLEAR

1. Concerns regarding applicability

Is there concern that the index test, its conduct, or interpretation differ from the review question?

CONCERN: LOW /HIGH/UNCLEAR

**DOMAIN 3: REFERENCE STANDARD**

1. Risk of Bias

Describe the reference standard and how it was conducted and interpreted:

Is the reference standard likely to correctly classify the target condition?

Yes/No/Unclear

Were the reference standard results interpreted without knowledge of the results of the index test?

Yes/No/Unclear

Could the reference standard, its conduct, or its interpretation have introduced bias?

RISK: LOW /HIGH/UNCLEAR

1. Concerns regarding applicability

Is there concern that the target condition as defined by the reference standard does not match the review question?

CONCERN: LOW /HIGH/UNCLEAR

**DOMAIN 4: FLOW AND TIMING**

1. Risk of Bias

Describe any patients who did not receive the index test(s) and/or reference standard or who were excluded from the 2x2 table (refer to flow diagram):

Describe the time interval and any interventions between index test(s) and reference standard:

Was there an appropriate interval between index test(s) and reference standard?

Yes/No/Unclear

Did all patients receive a reference standard?

Yes/No/Unclear

Did patients receive the same reference standard?

Yes/No/Unclear

Were all patients included in the analysis?

Yes/No/Unclear

Could the patient flow have introduced bias?

RISK: LOW /HIGH/UNCLEAR
